# Tailor-Made Fluorinated Ionic Liquids for Protein Delivery

**DOI:** 10.3390/nano10081594

**Published:** 2020-08-14

**Authors:** N. S. M. Vieira, P. J. Castro, D. F. Marques, J. M. M. Araújo, A. B. Pereiro

**Affiliations:** LAQV, REQUIMTE, Departamento de Química, Faculdade de Ciências e Tecnologia, Universidade Nova de Lisboa, 2829-516 Caparica, Portugal; ns.vieira@campus.fct.unl.pt (N.S.M.V.); pj.castro@fct.unl.pt (P.J.C.); daniela.marques019@gmail.com (D.F.M.); jmmda@fct.unl.pt (J.M.M.A.)

**Keywords:** fluorinated ionic liquids, aggregation behavior, delivery systems, biological activity, encapsulation efficiency, release

## Abstract

Nowadays, pharmaceutical companies are facing several challenges with the development and approval of new biological products. The unique properties of several fluorinated ionic liquids (FILs), such as their high surfactant power in aqueous solutions, their chemical and biological stability, and low toxicity, favor their application in the pharmaceutical industry. Furthermore, the numerous combinations between cations and anions, in the FILs design, enlarge the possibilities to construct a successful delivery system. Several FILs also proved to not affect the activity, stability, and secondary structure of the therapeutic protein lysozyme. This work aims to study the aggregation behavior of distinct FILs in the protein suitable medium, in the presence or absence of lysozyme. Besides, different incubation conditions were tested to guarantee the optimal enzymatic activity of the protein at more stable delivery systems. Following the optimization of the incubation conditions, the quantification of the encapsulated lysozyme was performed to evaluate the encapsulation efficiency of each FIL-based system. The release of the protein was tested applying variables such as time, temperature, and ultrasound frequency. The experimental results suggest that the aggregation behavior of FILs is not significantly influenced by the protein and/or protein buffer and supports their application for the design of delivery systems with high encapsulation efficiencies, maintaining the biological activity of either encapsulated and released protein.

## 1. Introduction

Biological products with therapeutic properties, such as peptides and proteins, exhibit increased safety, target specificity, and favorable pharmacokinetics when compared to other conventional drugs [1,2,3,4]. Additionally, these biological compounds are usually used to treat several diseases that are not treatable with other traditional drugs [1,2,3,4]. Almost 30% of all compounds approved by the Food and Drug Administration (FDA) between 2015 and 2018 are biologics, with 150 peptide drugs in clinical trials [5,6]. Furthermore, the biologics market is expected to reach 625.6 million dollars by the year of 2026, which represents a great opportunity for pharmaceutical industries [7].

Several types of protein-based therapeutics, such as the ones based on monoclonal antibodies, enzymes, hormones, growth factors, vaccines, anticoagulants, blood factors, and thrombolytics are in development stage or are already in the market [8,9]. However, the administration of these biomolecules through the conventional routes is hindered by: (a) their short half-life in the blood circulation; (b) their high-molecular-weight that limits their absorption in the blood circulation; (c) their biological nature that favors their enzymatic degradation in the gastrointestinal tract; and (d) their chemical and physical instability [10]. To overcome these problems without compromising the therapeutic effect of the biomolecules, high drug doses are usually administrated, which may generate several hostile side effects [11].

The design of drug delivery systems (DDSs) for therapeutic proteins appears as a promising option to circumvent the problems associated to the traditional routes of drug administration (oral and intravenous). The incorporation of the biomolecules in the DDSs prevents their enzymatic degradation and allows a time- and site-specific release, which ends in a reduced effective drug dosage needed. Despite the boost in the number of studied DDSs based on liposomes, microparticles, nanoparticles, among others, there are still considerable limitations in the development of efficient DDSs [12,13,14]. The low stability associated to some lipidic formulations, the uncontrolled release of the proteins, and the low encapsulation efficiencies are amongst the most common drawbacks of traditional DDSs [12,13,14]. Therefore, there is an urge to design new, innovative, and efficient DDSs for the optimal delivery of therapeutic proteins.

The use of ionic liquids (ILs) for biomedical and pharmaceutical applications has increased in the last years [15]. ILs have been conceived either as active pharmaceutical ingredients (API) itself or as dispersion agents, catalysts, or reaction media for the design of DDSs [15,16]. Moreover, ILs can also be used in the development and optimization of biomedical analytical techniques, such as electrochemical sensors, in mass spectrometry, in nuclear magnetic resonance (NMR), in chromatography, and in extraction processes, among others [15].

This work is focused on a poorly explored family of ILs containing fluorine tags with at least four carbon atoms, the fluorinated ionic liquids (FILs). This family combines several and unique properties inherent to traditional fluorinated surfactants and to ionic liquids, such as chemical and biological inertness, low vapor pressure, high thermal and chemical stability, low surface tension, and ability to form stable self-assembled structures [17,18,19,20,21,22]. By playing with the cation and anion core, as well as changing the length of the alkyl and fluorinated chains, it is possible to obtain FILs with a complete water miscibility, with hydrophilic behavior, low toxicity in different human cells (intestinal (Caco-2), hepatic (HepG2), endothelial (EA.hy926), and skin (HaCaT)), and almost negligible ecotoxicity towards different aquatic microorganisms [17,20,22,23,24]. These characteristics are in clear contrast to the persistent, bioaccumulative, and toxic nature of several traditional fluorinated compounds [25,26,27,28,29,30,31]. The FILs studied in this work were all based on the perfluorobutanesulfonate anion, a conjugate base of perfluorobutanesulfonate acid (applied in several industrial fields as substitute to long chain perfluoro alkyl acids). Contrarily to the long-chain perfluoro alkyl acids, these short-chain-based substituents are not considered bioaccumulative and have a shorter half-life [25,26,27,28,29,30,31]. Several toxic effects were associated to the exposure to perfluoro compounds with long chain lengths, including reduced intercellular communication, inhibited cell proliferation, toxic responses in different cells, neurotoxicity, endocrine disruption, and carcinogenesis, among others [25,27,28,29,30]. These toxic effects were much less pronounced or null in perfluorobutanesulfonate substituents, which have also been proved to not induce an oxidative damage in HepG2 cells and have a very low toxicity to mammalian cells, birds, algae, invertebrates, fish, and sewage organisms [26,27,31]. This profile is in line with the results of cytotoxicity and ecotoxicity studies performed with FILs based on the perfluorobutanesulfonate anion [17,23,24]. The resistance of these compounds to degradation, either by hydrolysis or photolysis, is a concern limited by their reduced bioaccumulation (bioconcentration factor <1) and low toxicity [26,27]. The information herein collected reinforces the relevant role of FILs as innovative and safe components in the pharmaceutical industry.

Recently, FILs based on imidazolium, pyridinium, and cholinium cations, conjugated with the perfluorobutanesulfonate anion, were tested for the encapsulation of lysozyme [32]. These FILs were selected based on a previous screening on their thermophysical, surfactant, cytotoxic, and ecotoxic properties [17,18,19,20,21,22,23,24]. In this previous work, it was proved that the activity, stability, and secondary structure of lysozyme was not affected up to concentrations three times higher than the first critical aggregation concentration of the selected FILs [32]. Furthermore, the encapsulation of the protein was analyzed and confirmed by dynamic light scattering (DLS) and microscopy studies [32]. To our knowledge, this work is the first study showing advances in the application of FILs as delivery agents for therapeutic proteins [32]. Therefore, we stepped forward in the study of FILs-based delivery systems with lysozyme by investigating several topics, such as the aggregation behavior of FILs in protein medium, in the presence and absence of lysozyme; the biological activity of the protein at different incubation temperatures; the encapsulation efficiency of each FIL-based delivery system in the presence of different protein and FIL concentrations; the subsequent release of the protein from the delivery system after the application of several external stimuli, such as temperature, time, and the presence of ultrasounds; and finally, the biological activity of both encapsulated and released protein.

## 2. Materials and Methods 

### 2.1. Lysozyme and Fluorinated Ionic Liquids

Lyophilized lysozyme from chicken egg white, *Micrococcus lysodeikticus* lyophilized cells, was purchased from Sigma-Aldrich (St. Louis, MO, USA). Potassium phosphate monobasic (purity ≥ 99.0%) was purchased from Fluka (Charlotte, NC, USA) and Micro BCA™ and BCA™ Protein Assay Kit were purchased from Thermo Fisher Scientific (Waltham, MA, USA). Milli-Q water (obtained from a Milli-Q Integral water purification system from Merck, Darmstadt, Germany) was used for the buffer preparation.

1-Ethyl-3-methylimidazolium perfluorobutanesulfonate, [C_2_C_1_Im][C_4_F_9_SO_3_] (≥97% mass fraction purity), 1-ethyl-3-methylpyridinium perfluorobutanesulfonate, [C_2_C_1_py][C_4_F_9_SO_3_] (≥99% mass fraction purity), and cholinium ((2-hydroxyethyl)trimethylammonium) perfluorobutanesulfonate, [N_1112(OH)_][C_4_F_9_SO_3_] (≥97% mass fraction purity) were supplied by IoLiTec GmbH (Salzstraße, Heilbronn, Germany). To reduce volatile chemicals and water contents, all FILs were dried under vacuum (4 Pa) with vigorous stirring at about 50 °C for at least 2 days, immediately prior to their use. No further purification was carried out and the purity of all FILs was checked by ^1^H and ^19^F NMR. The chemical structures of the fluorinated ionic liquids used in this work are presented in Table 1.

### 2.2. Aggregation Behavior of Fluorinated Ionic Liquids

The aggregation behavior of FILs in 66 mM potassium phosphate buffer (pH = 6.2) was determined by the ionic conductivities measured through a titration method. The ionic conductivities were measured with a CDM210 conductometer (Radiometer Analytical, Lyon, France), using a CDC749 electrode (Radiometer Analytical, Lyon, France) in a glass cell with a magnetic stirrer at 25 °C. The equipment was calibrated for the selected temperature using a certified 0.01 D KCl standard solution, provided by Radiometer Analytical (Lyon, France). The cell was thermostatized with a water bath, and the temperature was registered using a platinum resistance thermometer coupled to a Keithley 199 system DMM/scanner (uncertainty of ±0.1 °C) from Keithley Instruments (Solon, OH, USA). The cell contained a solution of FIL in buffer with an initial concentration higher than the third critical aggregation concentration (previously determined in water) [20]. The sample was stirred after each addition and conductivity was measured. To evaluate the influence of the biomolecule in the aggregation behavior of FILs, an identical procedure was performed with an initial solution of FIL containing lysozyme at 0.2 mg/mL in buffer. Conductivity was measured at least three times, and the uncertainty of each measurement was estimated to be less than 1%.

### 2.3. Lysozyme Bioactivity 

The bioactivity of lysozyme (defined as its lytic activity against the cell wall of *Micrococcus lysodeikticus*) was evaluated in either the presence or the absence of FILs by following the changes in turbidity of the bacterial suspension. Absorbance at 450 nm was measured in flat-bottomed 96-well plates purchased from Nunc, Thermo Fisher Scientific (Waltham, MA, USA) in a Multiskan GO microplate reader also from Thermo Fisher Scientific (Waltham, MA, USA), as previously detailed [32,33,34]. 

Solutions containing lysozyme (0.2 mg/mL) and FILs (0.1, 0.6, 1.2, and 1.8% *v*/*v*) were prepared in 66 mM potassium phosphate buffer (pH = 6.2) and incubated at 4, 25, and 37 °C for 24 h. Solutions containing only lysozyme (0.2 mg/mL) or FILs (0.1, 0.6, 1.2, and 1.8% *v*/*v*) in buffer were prepared and incubated in the same conditions to be used as positive and negative controls, respectively. A 0.3 mg/mL substrate solution of *M. lysodeikticus* was prepared in the same protein buffer and was allowed to settle for at least 30 min. To evaluate the bioactivity after protein encapsulation, an aliquot of 100 µL of the *M. lysodeikticus* suspension was added to 10 µL of tested solutions containing lysozyme (0.05 mg/mL) in a 96-well plate at 25 °C. Final concentrations of 0.27 and 0.005 mg/mL were obtained for substrate and lysozyme solutions, respectively. Measurements of substrate in the absence of lysozyme were performed as blank assays. Absorbance at 450 nm was measured immediately after substrate addition and was monitored at 30 s intervals for 5 min. The measurements were made in triplicates in, at least, two independent experiments. The A_450_ values at each time point were plotted and used to determine the linear turbidity decline (slope) and the biomolecule activity.

### 2.4. Lysozyme Encapsulation Efficiency

Solutions containing lysozyme (0.04 and 0.2 mg/mL) and FILs (0.1, 0.6, 1.2, and 1.8% *v*/*v*) were prepared in 66 mM potassium phosphate buffer (pH = 6.2) and incubated at 4 °C for 24 h. Solutions containing only lysozyme (0.04 and 0.2 mg/mL) or FILs (0.1, 0.6, 1.2, and 1.8% *v*/*v*) in buffer were prepared and incubated in the same conditions to be used as positive and negative controls, respectively. After incubation, the samples were centrifuged at 4 °C for 30 min at 10,000 rpm, to separate the unencapsulated lysozyme in solution (detected in the supernatant) from the encapsulated protein (detected in the pellet). Protein concentration was determined in both phases in flat bottom 96-well plates using the colorimetric microBCA and BCA Protein Assay Kits purchased from Thermo Fisher Scientific (Waltham, MA, USA) [35]. Buffer solutions, containing only lysozyme or FILs, were measured as positive and negative controls, respectively. The supernatant solutions were directly measured, by sampling 100 µL to the 96-well microplate. The pellets were firstly diluted in the initial volume and stirred until a homogeneous solution was obtained. Then, 100 µL of these samples were added to the microplate. A set of known concentrations of lysozyme in buffer were used as standards. The final lysozyme concentration was determined through the polynomial equation obtained through the standard curve. The measurements were made in triplicates in, at least, two independent experiments. The following equation was used to estimate lysozyme encapsulation efficiency in the different studied FILs:

(1) $$Encapsulation\;efficiency\;\left(\%\right)=\frac{Encapsulated\;amount\;of\;lysozyme}{Total\;amount\;of\;lyzozyme\;}\times 100\%$$

### 2.5. In Vitro Release of Lysozyme from FILs Delivery Systems

After the initial incubation process, several external stimuli, such as time, temperature, and ultrasounds, were employed at the sample. Then, the amount of protein released from FILs nanostructures was measured by BCA Protein Assay Kit (Thermo Fisher Scientific, Waltham, MA, USA) [35], in a procedure similar to the described in Section 2.3. Protein release was evaluated at different time points (t = 3, 6, 8, and 12 h) at 4 and 37 °C, as well as after 1 h at 42 °C. Additionally, the samples were also placed in an ultrasound bath for 1 h at the frequency of 80 kHz. Finally, the bioactivity of the released protein was evaluated using the same experimental procedure described in Section 2.3. The measurements were made in triplicates in, at least, two independent experiments.

## 3. Results

### 3.1. Aggregation Behavior of Fluorinated Ionic Liquids

**Figure 1:**
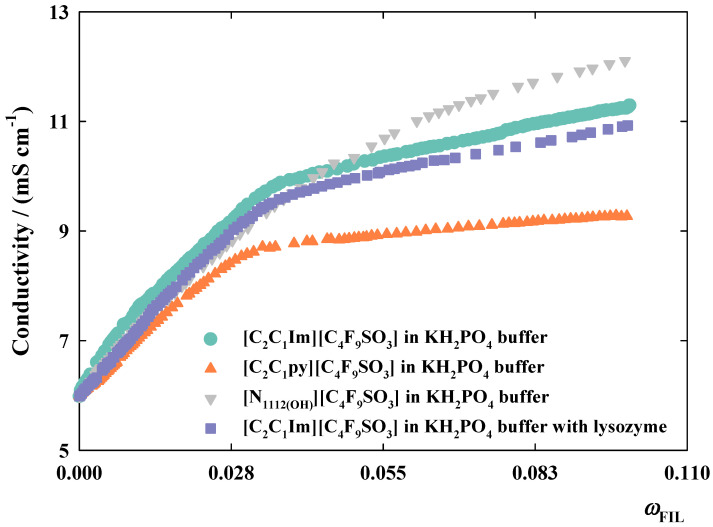
Conductivity profiles of [C_2_C_1_Im][C_4_F_9_SO_3_]—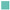, [C_2_C_1_py][C_4_F_9_SO_3_]—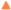, and [N_1112(OH)_][C_4_F_9_SO_3_]—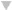 in 66 mM KH_2_PO_4_ buffer (pH = 6.2) and [C_2_C_1_Im][C_4_F_9_SO_3_]—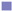 in the same buffer with lysozyme (0.2 mg/mL).

The selected fluorinated compounds used in this work are totally miscible in water and present a self-aggregation behavior dependent on their concentration. This self-aggregation behavior presents two distinct profiles in which the increment of FIL concentration is firstly associated to an increment of conductivity, followed by a decline on this property. In diluted solutions, the increment of conductivity is explained by the relatively higher number of free ions in solution. This linear behavior is not observed when higher concentrations of FILs are being tested, mainly due to the lower fluidity and mobility of the ions, which starts to be reduced when the aggregation process begins [20]. In this work, the aggregation behavior of each FIL was studied in 66 mM potassium phosphate buffer (pH = 6.2) by measuring the ionic conductivity at different FIL concentrations at 25 °C, as depicted in Table 2 and illustrated in Figure 1. Moreover, the influence of lysozyme in the aggregation behavior of [C_2_C_1_Im][C_4_F_9_SO_3_] in this buffer solution was also investigated. The corresponding critical aggregation concentrations (CACs) were determined using Phillips definition and based on the sharp change in the slope depicted in plots. Then, these values were compared with the ones previously obtained in pure water [20] to evaluate the influence of the buffer and the protein in the aggregation behavior of FILs.

Although the pH of pure water and of the tested buffer were similar, the electrolyte concentration can influence the final CAC values, as already mentioned in literature for other ionic liquids and traditional surfactants [36,37,38]. However, in the presence of the phosphate buffer, slight differences were noticed at the second CAC for [N_1112(OH)_][C_4_F_9_SO_3_] and [C_2_C_1_py][C_4_F_9_SO_3_], as well as in the third CAC of all FILs studied in this work. These small differences can be explained by the greater surfactant nature of these compounds in comparison to the traditional surfactants and ionic liquids [20]. As mentioned in previous works [18,20], FILs with only four carbon atoms in the fluorinated alkyl chain exhibit a better surfactant behavior than conventional perfluorosurfactants with eight carbon atoms chain and the corresponding hydrogenated counterparts. As depicted in Table 2, the differences in the aggregation of [C_2_C_1_Im][C_4_F_9_SO_3_] in pure water (0.0060 and 0.0158 *w*_FIL_) [20] and in buffer (0.0060 and 0.0157 *w*_FIL_) for first and second CAC, respectively, were null or insignificant. In the case of [C_2_C_1_py][C_4_F_9_SO_3_] and [N_1112(OH)_][C_4_F_9_SO_3_], the smallest variations in the aggregation behavior in buffer were obtained in the first CAC, as observed in Table 2. Furthermore, in these same FILs, the second transition seems to be less favored in the presence of the buffer solution, since the values for the second CAC increased (0.0130 and 0.0206 *w*_FIL_) in comparison to the values obtained in pure water (0.0119 and 0.0142 *w*_FIL_, respectively) [20]. In contrast, for the third transition, the values obtained in water were 0.0332, 0.0325, and 0.0751 *w*_FIL_, for [C_2_C_1_Im][C_4_F_9_SO_3_], [C_2_C_1_py][C_4_F_9_SO_3_], and [N_1112(OH)_][C_4_F_9_SO_3_] [20], whereas in buffer, they slightly dropped to 0.0319, 0.0284, and 0.0682 *w*_FIL_, respectively. This last transition can be slightly favored in the presence of buffer solution where the buffer ions allow a faster FIL aggregation. In the presence of lysozyme, the values for both first and third CAC of [C_2_C_1_Im][C_4_F_9_SO_3_] in buffer increase, which can be justified by the impact of the three-dimensional structure of the biomolecule in the FIL aggregation process. This impact may be different for the second transition which starts to occur at lower FIL concentrations. 

Albeit there are slight modifications in the determined aggregation concentrations of [C_2_C_1_Im][C_4_F_9_SO_3_], [C_2_C_1_py][C_4_F_9_SO_3_], and [N_1112(OH)_][C_4_F_9_SO_3_] in buffer and in the presence of lysozyme, the overall aggregation behavior of the FIL is not significantly affected, as can be confirmed by the determined CAC values depicted in Table 2, and by the conductivity profiles represented in Figure 1. The same tendency was verified in the aggregation behavior of [C_2_C_1_py][C_4_F_9_SO_3_] and [N_1112(OH)_][C_4_F_9_SO_3_] in 66 mM potassium phosphate (buffer pH = 6.2) where no significant variations were observed in comparison to that behavior in water [20].

### 3.2. Lysozyme Bioactivity 

**Figure 2:**
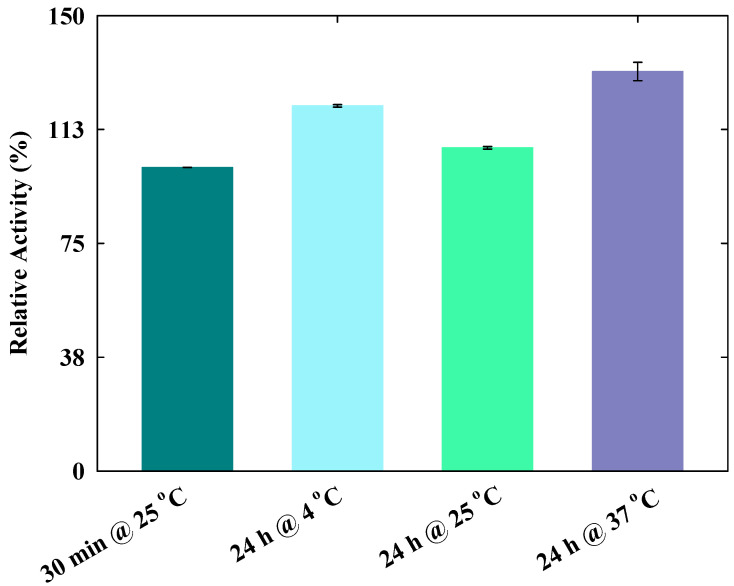
Lysozyme relative activity at different incubation conditions. The error bars indicate the standard deviations of the mean values.

The lytic activity of lysozyme in the presence of several FILs at different concentrations was tested to determine the best conditions for protein encapsulation without loss of its activity. The enzyme activity and stability are strongly dependent on several parameters, such as the salt concentration, the presence of specific ions, temperature, pH, and the preparation of the bacterial substrate [33,34,39,40]. To confirm that the tested incubation conditions do not affect the activity of the protein, a comparison study of the lysozyme activity under different incubation conditions without FILs was initially performed. Herein, the incubation at 25 °C for 30 min was taken as the reference for this specific test, because it was previously observed that the lysozyme activity is maintained after an incubation at room temperature for 30 min, in all ranges of tested FIL concentrations [32]. It was also observed before, through DLS, that a minimum incubation of 24 h leads to more stable structures containing both FILs and lysozyme [32]. Thus, in this study, the enzymatic activity of lysozyme was firstly tested at 25 °C, after an incubation for 24 h, at 4, 25, and 37 °C in the absence of FILs. The temperatures of 4 and 25 °C were selected based on different possible storage conditions, including refrigeration and a controlled temperature close to the room temperature, respectively. Furthermore, we also evaluated the activity of the protein after an incubation in a temperature identical to the average body temperature, 37 °C. The results, depicted in Figure 2, show that the protein activity was maintained after all tested time and incubation temperatures, 24 h at either 4, 25, and 37 °C, in the absence of FILs, with relative activity values higher than the reference.

**Figure 3:**
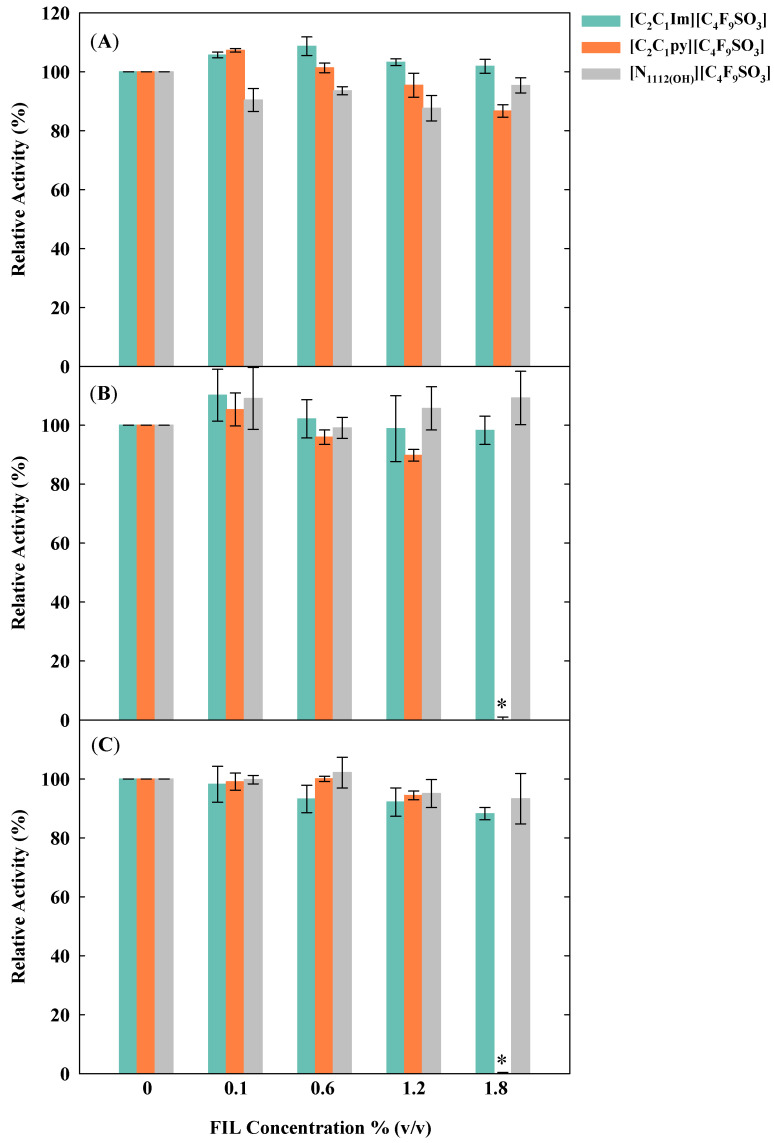
Lysozyme activity in different FILs concentrations at: (**A**) 4 °C, (**B**) 25 °C, and (**C**) 37 °C. * Under these conditions the lysozyme activity is null. The error bars indicate the standard deviations of the mean values.

Then, the bioactivity of the protein was evaluated in the presence of several concentrations of FILs at the different tested incubation conditions. The results are illustrated in Figure 3. The selected concentrations for this study (0.1, 0.6, 1.2, and 1.8% *v*/*v*) were chosen accordingly to the CACs of these compounds [20]. A concentration below the CAC, 0.1% *v*/*v*, was tested to confirm that in the absence of the FILs aggregates, the protein was not encapsulated and its activity was not affected by the FIL. Furthermore, FILs concentrations of 1.2 and 1.8% *v*/*v* were tested because these concentrations (higher than the CAC values) enhance the self-assembling of the micellar structures that allow the encapsulation of the protein [20,32].

Considering that the absorbance values for both substrate and FILs blanks remain constant along the 5 min, it was possible to neglect these values when determining the bioactivity of lysozyme. Furthermore, the lysozyme activity is not affected by the presence of ionic liquids (previously demonstrated with the A_450_ values) [32]. Herein, we calculate the activity of lysozyme from the slope of the time in which the turbidity decline is linear. These studies bring us insights about the best incubation temperature for the protein in more stable aggregates of FILs, as well as data about the temperature effect on the biomolecule activity. The activity of the sample containing pure lysozyme in buffer was used as reference (100% of activity) for each incubation condition with FILs. The results, presented in Figure 3, show that the overall optimal tested incubation condition was 24 h at 4 °C. Under these conditions the relative activity of the protein remains always above 86.7% for all tested FILs. Otherwise, for the incubation at higher temperatures (25 and 37 °C), there is a complete loss of lysozyme activity with [C_2_C_1_py][C_4_F_9_SO_3_] 1.8% *v*/*v* (see Figure 3B,C). The loss of activity at this [C_2_C_1_py][C_4_F_9_SO_3_] concentration at the highest temperatures may be caused by a modification on the permeability, interaction, or structure of the protein-FILs aggregates, since the activity of the free protein without FILs was not affected at these high temperatures, as shown in Figure 2.

### 3.3. Lysozyme Encapsulation Efficiency 

The lysozyme encapsulation efficiency (EE %) in [C_2_C_1_Im][C_4_F_9_SO_3_], [C_2_C_1_py][C_4_F_9_SO_3_], and [N_1112(OH)_][C_4_F_9_SO_3_] was evaluated at different FIL concentrations (0.1, 0.6, 1.2, and 1.8% *v*/*v*). These concentrations correspond to values before and after the first CAC of these compounds, which is approximately 0.6% *v*/*v*. A concentration below the CAC value, 0.1% *v*/*v*, was tested to confirm that in the absence of the FILs aggregates, the protein was not encapsulated. Then, the encapsulation efficiency at this FIL concentration is expected to be close to zero. Furthermore, 1.2 and 1.8% *v*/*v* (2 and 3 times higher than first CAC, respectively) were tested since self-assembling structures occur at concentrations higher than the CAC values which allow the encapsulation of the protein [32]. These encapsulation studies were performed with lysozyme concentrations of 0.04 and 0.2 mg/mL, using the colorimetric microBCA and BCA Protein Assay Kits (Thermo Fisher Scientific, Waltham, MA, USA) following the protocol described in experimental Section 2.3.

In this study, we aim to evaluate the effect of the FIL concentration, as well as the influence of the amount of protein in the encapsulation efficiency of each FIL-protein system. A centrifugation was performed to separate the encapsulated lysozyme (pellet) from the free lysozyme in solution (supernatant). When a pellet was detected, the amount of lysozyme was measured through the quantification of the biomolecule in both phases: supernatant and pellet. The concentration of lysozyme blank solutions (containing lysozyme at 0.04 and 0.2 mg/mL in buffer) was used as reference to determine the encapsulation efficiency of each FIL system. Samples containing only FILs were also used as negative controls. The amount of lysozyme detected in the supernatant in addition to the amount of protein determined in the pellet corresponds to the total amount of biomolecule used for the sample preparation. The EE % of each protein concentration was calculated for each FIL-based system. It is presented in Table 3.

The results show that the higher EE% were achieved at 1.8% (*v*/*v*), corresponding to concentrations three times higher than first CAC, where the aggregates are more stable. No significant differences were observed by increasing protein concentration from 0.04 to 0.2 mg/mL (see Table 3). Although the aggregation behavior is mainly driven by the fluorinated anion core of the FIL, which is identical in all tested systems, the cation seems to play an important role in the encapsulation of this protein. This influence is proved by the low EE% obtained with the nonaromatic cation, cholinium ([N_1112(OH)_]^+^), in comparison to both imidazolium ([C_2_C_1_Im]^+^) and pyridinium ([C_2_C_1_py]^+^)-based FILs. Further studies must be performed to understand the interaction between cholinium-based FILs and this biomolecule, which could explain the low encapsulation profile obtained with this specific cation. Although the results obtained with cholinium were not the expected, the encapsulation efficiencies with the other FILs at the higher concentration were all above 69.4%, with a maximum value of 83.4% achieved for [C_2_C_1_py][C_4_F_9_SO_3_]. These encapsulation efficiencies are relatively higher than others obtained with several traditional platforms such as microspheres and PLGA-based materials (ranging from 10.9% to 83%) [41,42,43,44] and very similar to the high entrapment rates (72–91%) of biomolecules in liposomes [45].

The amount of lysozyme initially tested has a small effect on the final encapsulation efficiency in the FIL based systems, with slightly better results obtained for the solutions containing 0.2 mg/mL of protein. Based on the absence of encapsulation for [N_1112(OH)_][C_4_F_9_SO_3_] in all tested concentrations, this FIL was discarded for the subsequent studies. Moreover, the same decision was taken for the FILs concentrations below 1.8% (*v*/*v*) for either [C_2_C_1_Im][C_4_F_9_SO_3_] or [C_2_C_1_py][C_4_F_9_SO_3_], where no pellet was observed, indicating the absence of encapsulation. Then, with the high encapsulation efficiencies demonstrated for 1.8% (*v*/*v*) in both imidazolium and pyridinium-based systems, the application of FILs as delivery systems in the pharmaceutical industry is possible. 

### 3.4. In Vitro Release of Lysozyme from FILs Delivery Systems

The low storage stability and the high initial burst release of drugs and biomolecules encapsulated in delivery platforms are huge limitations in the pharmaceutical development and must always be evaluated [46,47]. Based on the better results obtained for the encapsulation of lysozyme, this study was performed with solutions containing lysozyme at 0.2 mg/mL and [C_2_C_1_Im][C_4_F_9_SO_3_] and [C_2_C_1_py][C_4_F_9_SO_3_] at 1.8% *v*/*v*. To evaluate the stability of the delivery systems based on [C_2_C_1_Im][C_4_F_9_SO_3_] and [C_2_C_1_py][C_4_F_9_SO_3_] FILs stored at 4 °C, the amount of encapsulated lysozyme was measured at different time points (3, 6, and 12 h) after the incubation under the optimal conditions (24 h at 4 °C), using the colorimetric BCA Protein Assay Kits (Thermo Fisher Scientific, Waltham, MA, USA) following the protocol described in experimental Section 2.4.

By determining the amount of encapsulated lysozyme, it was possible to follow the leakage of the protein from the FIL-based delivery system, and consequently the stability of the delivery system over time. The results obtained are depicted in Table 4 and show that there is no significant release of the protein from the FIL delivery system up to 12 h postincubation. These results can be linked to the high stability of the fluorinated compounds that enhance and promote the retention of the biomolecule inside the FIL aggregates, as well as by the high encapsulation efficiencies obtained that promote higher interaction of the biomolecule with the FIL-based delivery platform.

**Figure 4:**
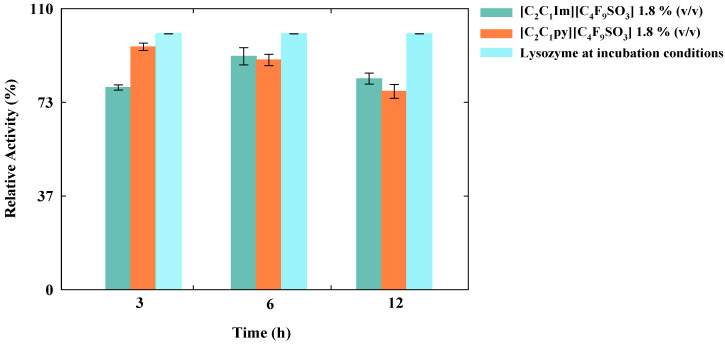
Relative activity of the encapsulated lysozyme (0.2 mg/mL) in [C_2_C_1_Im][C_4_F_9_SO_3_] and [C_2_C_1_py][C_4_F_9_SO_3_] after 3, 6, and 12 h after incubation at 4 °C during 24 h.

To consider any material as a delivery system, the release of the biomolecule should not occur in the storage conditions. In our study, the lysozyme release is not achieved under the specified incubation conditions, which is an advantage regarding the storage of these delivery systems. Furthermore, the relative activity of the protein was evaluated at these different time points (until 12 h post incubation at 4 °C). The results are illustrated in Figure 4. After centrifugation, the pellet was resuspended in the same initial volume and a sample was taken for the bioactivity assays. It was possible to confirm that the relative activity of the encapsulated lysozyme in the FIL-based delivery systems was always higher than 77.5% when compared to the relative activity of the lysozyme solution incubated, 24 h at 4 °C.

Furthermore, the release of the protein after the application of several external stimuli was analyzed using the colorimetric BCA Protein Assay Kit (Thermo Fisher Scientific, Waltham, MA, USA) following the protocol already mentioned. To better simulate the average body temperature and to predict the behavior of these systems in this condition, we evaluate the release of the protein after 6, 8, and 12 h at 37 °C. These time points were selected accordingly to standardized dosing times for therapeutics. After centrifugation, no pellet was observed which indicates the total release of the protein from the FIL-based delivery system. To confirm this result, the protein at the supernatant was quantified. The results are shown in Table 5.

The results show that the temperature increment from 4 to 37 °C causes a significant release of the protein from both [C_2_C_1_Im][C_4_F_9_SO_3_]- and [C_2_C_1_py][C_4_F_9_SO_3_]-based delivery systems. Approximately, 96% of the encapsulated biomolecule was released from [C_2_C_1_Im][C_4_F_9_SO_3_] aggregates after 12 h. In the case of [C_2_C_1_py][C_4_F_9_SO_3_], the total amount of encapsulated lysozyme was released after 6 h at 37 °C.

A similar approach was taken to evaluate the released protein after 1 h at 42 °C and after 1 h in an ultrasound bath with a frequency of 80 kHz. The results are depicted in Table 6. This temperature was selected to mimic a possible hyperpyrexia state associated to pathological condition, such as intracranial hemorrhage, sepsis, and severe viral infections, such as coronavirus disease 2019 [48,49]. As shown in Table 6, the amount of free lysozyme after this release is close to 66.2% and 49.5% for imidazolium- and pyridinium-based systems, respectively, which means that half of the biomolecule is in solution after 1 h at 42 °C. These results suggest that 57% and 39% of the encapsulated protein was released from [C_2_C_1_Im][C_4_F_9_SO_3_]- and [C_2_C_1_py][C_4_F_9_SO_3_]-based delivery systems, respectively. This is a promisor result regarding the thermoresponse of these delivery systems, which could also be associated to a controlled delivery of the biomolecule. Finally, when the solutions are immersed in an ultrasound bath with a frequency of 80 kHz during 1 h, an almost complete release of the protein from the delivery systems was observed. This approach is a very promisor mechanism to be used in clinical administrations for a faster and, conceivably, local release of the therapeutic protein in several tissues.

**Figure 5:**
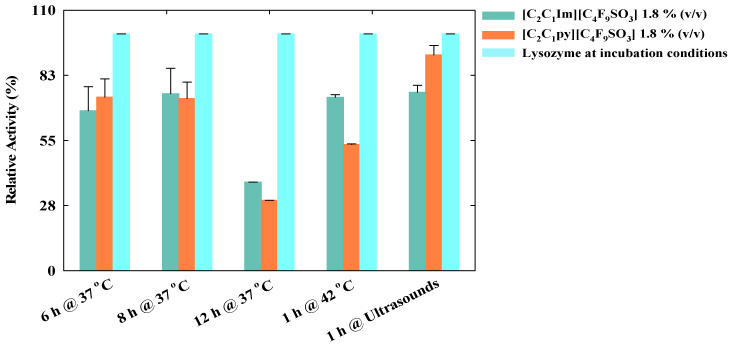
Relative activity of the released lysozyme (0.2 mg/mL) in [C_2_C_1_Im][C_4_F_9_SO_3_] and [C_2_C_1_py][C_4_F_9_SO_3_] after the exposure at several external stimuli.

The bioactivity of the released lysozyme was also tested after the application of these stimuli and the results are illustrated in Figure 5. When compared to the activity after incubation, the biomolecule retains its activity always above 50%, except for the longer incubation time at 37 °C. In this case, the biological activity of lysozyme dropped to the minimal values of 37.4% and 29.7% for [C_2_C_1_Im][C_4_F_9_SO_3_] and [C_2_C_1_py][C_4_F_9_SO_3_], respectively. However, this decrement on the activity is not a direct influence of the FIL, since the lysozyme blank placed at this condition has the same behavior, which means that for this longer period at 37 °C, the activity of lysozyme blank was also reduced. This could be caused by the temperature variations at which the samples were submitted, since after 24 h at 4 °C, the samples were placed more 12 h at 37 °C, which can affect the lysozyme activity. After the application of ultrasounds, the activity of the released protein is not significantly affected, which is a good indicator for a clinical approach.

## 4. Conclusions

The main purpose of this work was to evaluate the ability of FILs to be used for the design of drug delivery system for proteins with pharmacological properties. First, it was crucial to evaluate the preservation of the aggregation properties of FILs in the protein buffered medium, as well as in the presence of the biomolecule. Then, the better incubation conditions were determined evaluating the lytic activity of the protein after an incubation at different temperatures. After the optimization of the incubation conditions, the encapsulation efficiencies of different amounts of lysozyme in FILs at different concentrations were determined. Following the previous analysis, the stability of these systems was evaluated by measuring the release of the protein at different time points after incubation at 4 °C. Herein, the biological activity of the encapsulated protein was also measured. To evaluate the performance of these systems for the appropriate delivery of biomolecules, several external stimuli were applied, and the release of the encapsulated protein and its activity were determined.

The experimental results show that either protein or protein buffer have a small effect on the aggregation behavior of FILs. The conductivity measurements were compared with the ones already published in water, and the differences observed were not significant.

Based on the previous knowledge regarding the development of more stable structures of FILs-based delivery systems with lysozyme after 24 h of sample preparation, several temperatures of incubation were tested. It was demonstrated that after 24 h at 4 °C, the lytic activity of lysozyme was maintained for all FILs-based systems. This behavior was not observed at the higher tested temperatures in [C_2_C_1_py][C_4_F_9_SO_3_]. After the selection of the incubation conditions, the encapsulation efficiencies were determined for different FILs and protein concentrations. The results show that the biomolecule concentration does not influence the final encapsulation efficiency of FILs, which was higher for [C_2_C_1_py][C_4_F_9_SO_3_] and [C_2_C_1_Im][C_4_F_9_SO_3_] at 1.8% (*v*/*v*) in either 0.004 and 0.2 mg/mL of protein. Herein, it was demonstrated that the nonaromatic [N_1112(OH)_][C_4_F_9_SO_3_] do not encapsulate the protein even at the higher tested concentrations.

Based on the better results achieved for [C_2_C_1_py][C_4_F_9_SO_3_] and [C_2_C_1_Im][C_4_F_9_SO_3_], these FILs were selected for several release studies. First, no significant release of the protein occurs up to 12 h after incubation, proving the stability of the system. Then, to better simulate the physiological conditions of the human body, the samples were placed at 37 °C, and the release of the protein was evaluated at several time points, reaching the complete release after 12 h at this temperature.

Therefore, different external stimuli were applied, such as a temperature of 42 °C to mimic a pathological state, as well as an ultrasound frequency of 80 kHz for 1 h, which can be used as an alternative administration. After 1 h at 42 °C, approximately half of the protein was in solution in both systems, with slightly better results for [C_2_C_1_Im][C_4_F_9_SO_3_], in which the free protein reached values close to 66%, corresponding to a release of 57% of the initially encapsulated protein. Even so the better results regarding the release of the protein were obtained after the application of the ultrasounds, in which more than 95.8% of the protein was detected in solution for both systems. Moreover, the biological activity of the released protein was also evaluated, and a reduction higher than 50% after 12 h at 37 °C was noticed in both FILs. However, for the other tested stimuli, the activity of the protein was always above 50%, with values close to the maximum for [C_2_C_1_py][C_4_F_9_SO_3_] after the ultrasound-driven release.

The overall conclusions of this work strengthen the possibility for the application of biocompatible FILs as delivery systems for complex drugs, such as biomolecules. The high stability associated to the fluorinated compounds proved to be an advantage for the storage of the [C_2_C_1_Im][C_4_F_9_SO_3_]- and [C_2_C_1_py][C_4_F_9_SO_3_]-based delivery systems, without affecting the biological activity of the biomolecule. Moreover, the release of the protein was not impaired, and several external stimuli can be used. Then, the development of FIL-based delivery systems appears as a promisor alternative to the traditional paths for the delivery of therapeutic proteins.

## Figures and Tables

**Table 1:** Chemical structure and acronyms of the fluorinated ionic liquids (FILs) used in this work.

FIL Designation	Chemical Structure
1-Ethyl-3-methylimidazolium perfluorobutanesulfonate [C_2_C_1_Im][C_4_F_9_SO_3_]	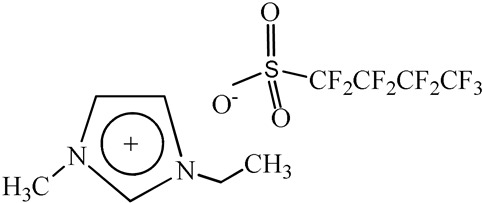
1-Ethyl-3-methylpyridinium perfluorobutanesulfonate [C_2_C_1_py][C_4_F_9_SO_3_]	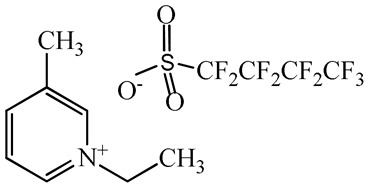
Cholinium ((2-hydroxyethyl)trimethylammonium) perfluorobutanesulfonate [N_1112(OH)_][C_4_F_9_SO_3_]	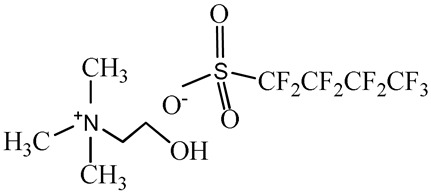

**Table 2:** Critical aggregation concentrations, CAC, determined by conductometry at 25 °C for [C_2_C_1_Im][C_4_F_9_SO_3_], [C_2_C_1_py][C_4_F_9_SO_3_], and [N_1112(OH)_][C_4_F_9_SO_3_] in different aqueous solutions.

		KH_2_PO_4_ Buffer	KH_2_PO_4_ Buffer with Lysozyme	Water [20]
[C_2_C_1_Im][C_4_F_9_SO_3_]				
First CAC	***w*_FIL_**	0.0060	0.0067	0.0060
Second CAC	***w*_FIL_**	0.0157	0.0136	0.0158
Third CAC	***w*_FIL_**	0.0319	0.0347	0.0332
[C_2_C_1_py][C_4_F_9_SO_3_]				
First CAC	***w*_FIL_**	0.0063	n.a.	0.0058
Second CAC	***w*_FIL_**	0.0130	n.a.	0.0119
Third CAC	***w*_FIL_**	0.0284	n.a.	0.0325
[N_1112(OH)_][C_4_F_9_SO_3_]				
First CAC	***w*_FIL_**	0.0063	n.a.	0.0065
Second CAC	***w*_FIL_**	0.0206	n.a.	0.0142
Third CAC	***w*_FIL_**	0.0682	n.a.	0.0751

**Table 3:** Lysozyme encapsulation efficiency (%) in [C_2_C_1_Im][C_4_F_9_SO_3_], [C_2_C_1_py][C_4_F_9_SO_3_], and [N_1112(OH)_][C_4_F_9_SO_3_] at different concentrations in buffer.

Lysozyme (0.04 mg/mL)				
	**0.1% (*v*/*v*)**	**0.6% (*v*/*v*)**	**1.2% (*v*/*v*)**	**1.8% (*v*/*v*)**
**[C_2_C_1_Im][C_4_F_9_SO_3_]**	*	*	*	69.4 ± 2.80
**[C_2_C_1_py][C_4_F_9_SO_3_]**	*	*	*	80.9 ± 1.05
**[N_1112(OH)_][C_4_F_9_SO_3_]**	*	*	*	*
**Lysozyme (0.2 mg/mL)**				
	**0.1% (*v*/*v*)**	**0.6% (*v*/*v*)**	**1.2% (*v*/*v*)**	**1.8% (*v*/*v*)**
**[C_2_C_1_Im][C_4_F_9_SO_3_]**	*	*	*	78.7 ± 6.17
**[C_2_C_1_py][C_4_F_9_SO_3_]**	*	*	*	83.4 ± 3.01
**[N_1112(OH)_][C_4_F_9_SO_3_]**	*	*	*	*

* After centrifugation, no pellet was visually detected. Mean values calculated from two independent assays and their standard deviations.

**Table 4:** Amount of encapsulated lysozyme (0.2 mg/mL) (%) in [C_2_C_1_Im][C_4_F_9_SO_3_]- and [C_2_C_1_py][C_4_F_9_SO_3_]-based delivery systems after 3, 6, and 12 h at 4 °C.

	3 h	6 h	12 h
[C_2_C_1_Im][C_4_F_9_SO_3_] 1.8% (*v*/*v*)	76.0 ± 6.81	74.7 ± 8.58	80.9 ± 2.72
[C_2_C_1_py][C_4_F_9_SO_3_] 1.8% (*v*/*v*)	86.8 ± 4.59	82.5 ± 3.70	78.7 ± 6.36

Mean values calculated from two independent assays and their standard deviations.

**Table 5:** Amount (%) of free lysozyme (0.2 mg/mL) released from [C_2_C_1_Im][C_4_F_9_SO_3_]- and [C_2_C_1_py][C_4_F_9_SO_3_]-based delivery systems after 6, 8, and 12 h at 37 °C.

	6 h	8 h	12 h
[C_2_C_1_Im][C_4_F_9_SO_3_] 1.8% (*v*/*v*)	94.4 ± 4.23	93.3 ± 9.69	97.1 ± 8.98
[C_2_C_1_py][C_4_F_9_SO_3_] 1.8% (*v*/*v*)	99.4 ± 12.6	101 ± 0.390	109 ± 7.54

Mean values calculated from two independent assays and their standard deviations.

**Table 6:** Amount (%) of free lysozyme (0.2 mg/mL) released from [C_2_C_1_Im][C_4_F_9_SO_3_]- and [C_2_C_1_py][C_4_F_9_SO_3_]- based delivery systems after different stimuli factors.

	1 h at 42 °C	1 h at Ultrasounds, 80 kHz
[C_2_C_1_Im][C_4_F_9_SO_3_] 1.8% (*v*/*v*)	66.2 ± 9.07	95.8 ± 4.19
[C_2_C_1_py][C_4_F_9_SO_3_] 1.8% (*v*/*v*)	49.5 ± 0.744	106 ± 9.29

Mean values calculated from two independent assays and their standard deviations.
